# Health inequality implications from a qualitative study of experiences of poverty stigma in Scotland

**DOI:** 10.1016/j.socscimed.2019.04.033

**Published:** 2019-07

**Authors:** Greig Inglis, Fiona McHardy, Edward Sosu, John McAteer, Hannah Biggs

**Affiliations:** a Scottish Collaboration for Public Health Research and Policy, University of Edinburgh, 20 West Richmond Street, Edinburgh, EH8 9DX, UK; b The Poverty Alliance, UK; c University of Strathclyde, UK

**Keywords:** Scotland, stigma, Health inequalities, Poverty

## Abstract

**Rationale:**

Individuals living in Scotland's most deprived communities experience a higher burden of morbidity and early mortality than those living in more affluent areas. Experiences of poverty-based stigma may be one psychosocial mechanism through which socioeconomic position influences health, although there is little available data on this issue from a Scottish perspective.

**Objective:**

The aim of this study was to identify which aspects of poverty stigma are particularly salient to individuals with lived experience of poverty, and may therefore contribute to health inequalities.

**Methods:**

Five focus groups were conducted with 39 individuals with experience of living on low incomes in Scotland in order to explore their experiences and perceptions of poverty stigma.

**Results:**

Five main themes were identified, reflecting aspects of poverty stigma operating at various structural, public and individual levels: media representations of poverty; negative encounters with social security systems; perceived public attitudes regarding poverty in Scotland; lowered self-esteem and internalisation of negative attitudes, and; emotional responses to stigma.

**Conclusion:**

These dimensions of stigma potentially influence public health and health inequalities in Scotland, although future research will be necessary to quantify these and estimate their relationships with health outcomes.

## Highlights

• Relatively little is currently known about poverty-based stigma in Scotland.
• People with experience of poverty discussed this issue in five focus groups.
• Participants' accounts reflected several forms of poverty stigma.
• Poverty stigma operates at various structural, social, and individual levels.
• Poverty stigma is a potentially important determinant of health inequalities.

## 1 Introduction

Approximately 19 percent of the Scottish population is estimated to be living in poverty (Scottish Government, 2018). Socioeconomic position (SEP) is a key determinant of health, where individuals from lower socioeconomic backgrounds typically experience poorer health and longevity than more affluent individuals. Inequalities in premature morality by SEP in Scotland and internationally (McCartney et al., 2017) most starkly demonstrate this contrast between socioeconomic groups. In Scotland currently, premature mortality is 3.7 times higher in the most deprived areas compared to the most affluent areas (Scottish Government, 2017). Reducing health inequalities has been a priority for policy makers for almost four decades, although Scotland still has the widest health inequalities in Western Europe (Popham and Boyle, 2011). There is therefore a pressing need to better understand the causes of health inequalities in Scotland, in order to identify areas for action. The theory of fundamental causes holds that SEP confers a health advantage because individuals of higher SEP possess the resources necessary – such as income, wealth and power – to engage in health promoting strategies and to avoid a wide array of health risks (Phelan et al., 2010). The causes of health inequalities are therefore seen to be primarily structural, which in turn influence health through a wide range of more proximal environmental, social, psychological, and biological mechanisms (Adler and Stewart, 2010; McCartney et al., 2013).

Poverty-based stigma represents a range of psychosocial pathways through which SEP may affect health and therefore contribute to health inequalities (Fuller-Rowell et al., 2012; Kessler et al., 1999; Reutter et al., 2009). Link and Phelan (2001) define stigma as the co-occurrence of several psychosocial processes, where individuals are first labelled as possessing some socially significant characteristic and are subsequently associated with negative stereotypes. As a result, labelled persons experience discrimination and a loss of social status that leads to unequal outcomes for stigmatised groups. Stigma is a broad construct and covers many distinctive processes that operate at different levels. At the individual level, stigma can be conceptualised as the experiences and perceptions of stigmatised groups, such as beliefs regarding the extent to which others hold stigmatising attitudes; expectations and experiences of encountering stigma or discrimination; and the internalisation of negative stereotypes. At the public level, stigma largely relates to social attitudes and stereotypes toward groups, whilst the structural level concerns social policies and institutional practices that – either intentionally or unintentionally– restrict individuals’ opportunities, resources, or wellbeing (Corrigan et al., 2004; Pescosolido and Martin, 2015).

Stigma is a major social determinant of population health and has been highlighted as a fundamental cause of health inequalities (Hatzenbuehler et al., 2014). Reviews covering a range of stigmatised identities have found experiences of stigma to predict relatively poor physical (e.g. risk factors for cardiovascular disease, various physical conditions, self-reported health), as well as both positive (e.g. self-esteem, life satisfaction, well-being) and negative (e.g. depression, anxiety, negative affect) mental health outcomes (Mak et al., 2007; Pascoe and Smart Richman, 2009; Schmitt et al., 2014). As with SEP, the effects of stigma on health are mediated by a range of psychosocial processes, including depleted personal and material resources, increased stress, and maladaptive forms of emotion regulation and coping behaviours (Hatzenbuehler et al., 2014).

Several qualitative studies conducted within the UK (Chase and Walker, 2012; Walker, 2014) and internationally (Reutter et al., 2009; Sutton et al., 2014; Walker et al., 2013) demonstrate how individuals living on low incomes experience various forms of poverty-based stigma. Chase and Walker (2012) highlighted experiences of poverty-based shame, which arise from several processes such as the overt actions of others that cause individuals to feel devalued; perceptions of being judged negatively by others; and an internalised sense of failure as a result of their financial situation. Moreover, these experiences of shame appear to be ubiquitous across different countries and contexts (Walker et al., 2013). Several international studies suggest that such experiences may be associated with poorer health outcomes. In the United States for example, experiences of poverty and SEP-based stigma have been found to predict various markers of relatively poor mental health, including depressive symptoms, low self-esteem, and feelings of social anxiety and shame (Mickelson and Williams, 2008; Ren et al., 1999; Simons et al., 2017). Further still, one recent study reported that SEP-based discrimination predicts relatively poor sleep quality amongst African Americans (Van Dyke, Vaccarino, Quyyumi and Lewis, 2016). These data support the view that poverty stigma may at least partially mediate the relationship between SEP and health. A separate line of evidence further demonstrates that general measures of perceived discrimination – not specifically related to poverty or SEP – partially mediate socioeconomic inequalities in psychological distress among adults (Kessler et al., 1999) and allostatic load among adolescents (Fuller-Rowell et al., 2012). Prospective cohort studies of adults in the UK have similarly found perceptions of being treated unfairly to predict metabolic syndrome approximately six years later (De Vogli, Brunner & Marmot, 2007), as well as coronary events and general physical and mental health approximately 11 years later (De Vogli, Ferrie, Chandola, Kivimäki & Marmot, 2007). A further prospective study of American adults showed that perceived discrimination partially mediates the relationship between SEP recorded at baseline and self-rated health reported 17–19 years later (Fuller-Rowell et al., 2018).

Poverty-based stigma may therefore be an important determinant of health inequalities in Scotland, although there are few studies that explicitly test this hypothesis. There is a pressing need to better understand how stigma may contribute to population health in Scotland in particular, considering the large discrepancies in health experienced by citizens living in Scotland's most and least affluent areas. Given the breadth and complexity of stigma as a concept (Pescosolido and Martin, 2015), it is first necessary to identify the aspects of poverty stigma that are most salient to communities with lived experience of poverty in Scotland. The aim of the current study was therefore to explore how individuals with experience of living on a low-income in Scotland perceive and experience various forms of poverty stigma, with a view to understand how these experiences may affect health.

## 2 Method

### 2.1 Participants

In total, 39 participants took part in the study, recruited from community organisations supporting individuals living on low incomes, which were largely based in urban areas across central Scotland. Individuals were eligible to participate if they were aged 18 or over, were English speaking, and considered themselves to be living in poverty or on a low income. Individuals self-selected to take part in a focus group in response to advertisements distributed throughout the organisations that outlined the inclusion criteria. The average age of the sample was 42 years, while 24 (62%) participants were male and 14 were female (36%); one participant did not report gender. Regarding employment status, five participants (12%) were employed, six (15%) were unemployed, and 16 (41%) were unable to work due to illness or disability. Two participants (6%) were either in education or training, three (8%) were looking after home or family, and two (5%) were retired; five (13%) participants did not report their employment status. Most participants described themselves as White Scottish (67%), or either White British or White Irish (10%); nine (23%) participants did not report their ethnicity. Participants came from diverse backgrounds and represented various groups that are at the highest risk of experiencing poverty in Scotland, including individuals with long-term conditions, lone parents, and people with experience of homelessness.

### 2.2 Procedure

This qualitative study employed focus groups to provide rich insight into the lived experience of stigmatised groups. (Link et al., 2004). A semi-structured topic guide was created to explore how participants defined and understood poverty and stigma, before considering how those living on low incomes were regarded and treated by others in Scotland, such as situations in which people experiencing poverty might expect unfair treatment or discrimination, and the consequences of stigma for individuals and communities. Each focus group was facilitated by two researchers and lasted for approximately 1 h. Informed written consent was obtained from all participants at the outset of each focus group, and participants also received a £10 shopping voucher as a gesture of appreciation for taking part. Discussions were audio recorded and transcribed verbatim. The transcripts were then anonymised by replacing participants' names with pseudonyms. Ethical approval for this project was obtained from the Usher Research Ethics Group at the University of Edinburgh.

### 2.3 Analysis

Transcripts were entered into NVivo 10 and analysed using thematic analysis. Following the method described by Braun and Clarke (2006), each transcript was first read over by a researcher and an initial set of codes was generated and applied to the transcripts on subsequent readings. Codes that shared similar meanings were grouped together, and these groupings were reviewed and revised until the coded data had been organised into a set of internally consistent themes. The final set of themes were reviewed by another researcher familiar with the transcripts to check that they accurately reflected the content. Participants are referred to with pseudonyms throughout the reporting of the results.

## 3 Results

Five themes were identified through the thematic analysis: media representations of poverty; negative encounters with social security systems; perceived public attitudes regarding poverty in Scotland; lowered self-esteem and internalisation of negative attitudes; and emotional responses to stigma. Each theme conveys an aspect of stigma operating at either a structural, public, or individual level.

### 3.1 Perceptions and experiences of structural stigma

**Media representations of poverty.** Participants frequently discussed how the media represents low-income families and communities. These accounts largely concentrate on “benefits shows” and other television programmes that focus on the lives of individuals living on low incomes. Although these programmes are presented to the public as being factual, participants described how they promote highly negative and unrealistic representations and stereotypes of what it means to be living in poverty in the UK

*“I think most of the programmes about poverty are stereotyped anyway … Every programme that comes on that telly, you know what I mean, you've got slum landlords, slum tenants, and all the rest of it.* [Colin, focus group 3]

Participants described how these programmes portray benefit claimants as being undeserving of assistance (“*benefits scroungers, that's a media phrase isn't it?”* Michael, focus group 1), and portray unemployed people as failing to contribute to society. Participants also explained how benefits shows frequently portray the featured individuals as being financially irresponsible. According to one participant, for example, these programmes present low-income families as “*not really spending their money wisely,*” but instead as people who would “*rather spend it on drink than food and apply for every loan under the sun.*” [Susan, focus group 5].

Participants considered these programmes, and the stereotypes that they promote, as being highly influential in shaping public attitudes toward people experiencing poverty, and in particular, benefit claimants. In addition to these broader societal effects, benefits shows were also described as being harmful to the individuals and communities they feature. Participants perceived these programmes as being highly exploitative, where often-vulnerable groups are taken advantage of and at times ridiculed. As a result, individuals who take part in these shows were felt to be at risk of facing additional stigma from the public and a greater loss in social status.

*“… these people are probably in a very, very desperate situation, and they think that these shows are maybe gonna help them to get out of that. But then, they end up being perceived … worse than they were before.”* [Sally, focus group 3]

**Negative encounters with social security systems.** Participants’ experiences of claiming benefits and interacting with social security systems in the UK were often described as being degrading, punitive, and generally unsupportive. Participants described encountering stigma through the behaviour and attitudes of Job Centre staff for example (“*I think their attitude stinks*” [Carol, focus group 4]), who were described as often being critical, judgemental, or disinterested in helping claimants:

“*I think the way the Job Centres treat people who are on benefits is absolutely shocking. I mean, these are the people that are employed to help people to get back to work, but they're the most likely to judge you - you're not looking for enough jobs, you're not applying for enough jobs, you need to be doing more. And you're not gonna motivate people by constantly putting them down*.” [Linda, focus group 3]

Participants also reported several structural issues within the social security system that made it difficult for individuals to claim benefits. A recurring theme was that there is a lack of support available to claimants and a lack of guidance and information available to individuals concerning the benefits that they are eligible to claim. One participant, for example, described how *“it's as though it's a big secret”* [Paul, focus group 2]. In some cases, participants had received support from external representatives to help them access benefits, although there was a perception that individuals may not receive all the financial supports that they are entitled to when such support is not available.

“*… you've got representatives that are really good and that will say, no, I'll get you this money, I'll help you, and that's the only thing that keeps you going.*” [Emma, focus group 4].

Participants further explained how claimants are expected to meet certain conditions to receive out-of-work benefits. Such conditions were often described as being unfair or unrealistic, but they nevertheless carry a harsh penalty when they are not met, such as claimants’ benefits being temporarily revoked. In the following extract, two participants discuss being expected to perform 35 h of job searching each week to qualify for unemployment benefits, which was viewed as unrealistic given that available jobs may not change over days or weeks, and access to resources (computers, internet, etc) may vary:

“*I know it's like they're trying to motivate people to do as much as they can to look for work, but 35 hours a week … I can do that in 45 minutes, half an hour, and I can refresh the page, and it isn't gonna change.* [Graham, focus group 3]

*And people are struggling to get by, and some people haven't got internet and stuff to be doing all this job searching that they're expecting you to do. And then they'll take money off you, ‘cause you've not done enough”* [Gemma, focus group 3].

A final feature of perceived structural stigma within the social security system was evident from participants’ accounts of undergoing medical assessments to qualify for disability or sickness benefits. Participants recounted experiencing stigma and unfair treatment during these medicals and described feeling as though they were not being listened to, and that the validity of their health problems was frequently questioned or undermined. Based on these experiences, some participants argued that it was almost inevitable that individuals attending these medicals will be found to be fit for work, and that their sickness or disability benefits will be withdrawn:

*“… you're forced to go to a medical and prove that you are unfit for work and it's horrendous … some people have called this place in the city centre that you have to go Lourdes, because everyone that goes there is ill, but you come out according to them … cured … In other words, there's nothing wrong with you.*” [Scott, focus group 2].

Crucially, participants viewed the social security system as being deliberately designed to make the process of claiming benefits difficult, in order to reduce welfare spending and limit the number of claimants who receive their entitlements. Some participants further suggested that this may in turn deter people from claiming benefits altogether.

### 3.2 Perceptions of public stigma

**Perceived public attitudes regarding poverty in Scotland.** Aspects of perceived public stigma were apparent when participants discussed common social attitudes and stereotypes regarding low income communities. A common theme throughout these discussions was the view that individuals living in poverty are often blamed by others for their situation, where poverty is attributed to laziness or some other personal failing. Such explanations that frame poverty as an issue of personal responsibility were seen to be accepted by members of the public at the expense of alternatives that acknowledge the wider social or structural causes of inequalities. Claire for example, who is unable to work due to poor health, explains how individuals in her situation are perceived by others: “*They think you're just lazy and don't want to work* … [they] *don't understand that you can't work*” [focus group 4]. Dylan similarly describes how people working in low paid jobs are typically seen as “*not very intelligent, otherwise they would have gone to college or university*” [focus group 1]. Dylan goes on to argue however that “*that's not really true*” and presents a more structural explanation that is seemingly neglected in public discourse, specifically that low-paid workers have “*not had the opportunities*.”

Some participants discussed how public stigma can be place-based, where certain neighbourhoods are associated with negative stereotypes. Joanne for example, explained how others often look down on the area where she lives as being a “dive” and “scummy.” These stereotypes are further applied to the individuals who live there:

“ [people think that] *they'll turn to crime, and just start drinking and taking drugs, they won't go to college, they won't go to uni, they won't do anything with their life. Stuck in a dead end job, or something. I think people think they can't excel, they can't be better.*” [Joanne, focus group 3]

Participants viewed public stigma as stemming from both media representations of poverty and from a lack of public understanding about the causes of poverty. Similarly, public stigma was largley ascribed to individuals of relatively high socioeconomic status, who may not recognise the opportunities that they themselves have benefited from when making judgements about others. In the following extract, two participants, who are both unemployed, discuss how some groups who are privileged in their social connections assume that others do not work through choice.

“[some people have] *got jobs through people they know, like they haven't actually had to go out and get their own job*” [Hayley, focus group 5].

“*Aye, or it's been handed to them, like a business, or it's been left to them so they've been able to work all their days, and because you don't work, it's like, ‘oh, they don't want to work*’” [Susan, focus group 5].

### 3.3 Individuals’ responses to stigma

**Lowered self-esteem and internalisation of negative attitudes.** Participants frequently referred to the negative consequences of poverty stigma, and specifically, the effects that stigma can have on individuals' identities and sense of worth. In particular, being the target of negative public attitudes and stereotypes was seen to lead to feelings of devaluation, and lowered self-esteem. Tom [focus group 3] for example explained that, “*people's attitudes does knock your confidence, it does knock your self-esteem*”.

In some cases, participants also explained how public stigma can become internalised, and this process of self-stigmatisation was further seen to affect both individuals' self-esteem and self-efficacy. This concept is illustrated in the following example from Chris, who describes how internalised negative attitudes limit individuals’ aspirations and expectations of themselves, and subsequently their behaviour:

*“If somebody is calling you a waste of space, you're no good … through time you start believing that, [so] you're not going to apply for any half decent jobs, you will not go to college, you won't try and better yourself, because you believe from a young age … you're no good, so [you're] never going to amount to anything anyway, so why bother?”* [Chris, focus group 1]

When describing self-stigma, however, participants largely referred to how other people might internalise negative stereotypes and there was little evidence that participants had done so themselves. Instead, when participants spoke of themselves personally, they largely appeared to resist public stigma. For example, although Chris recognised the potential for others’ negative attitudes to become internalised in the previous example, he goes on to describe how he personally rejects stereotypes of benefits claimants being “scoungers”. In doing so, Chris is able to protect his social identity and self-esteem.

“*I don't take any notice of that* [negative public attitudes] *because I worked for years and years … when I see that or hear it, I don't put it to myself, although I'm on benefits at the moment, you know what I mean, but I don't really take it on board*” [Chris, focus group 1].

**Emotional responses to stigma.** Participants' accounts further detailed the emotional consequences of stigma. This largely referred to the effects of negative public attitudes, and a sense of being looked down on by others, which was described as leaving individuals feeling “horrible”, “rubbish”, and “depressed”. In the following extract, Jessica illustrates how she feels when she is labelled with the negative stereotypes that are commonly applied to the people who live in her neighbourhood. This extract further demonstrates that stereotypes don't have be internalised to provoke negative feelings:

“*It doesn't feel good, because I'm not, I know that I'm not like that, and my family aren't like that, you know? They're not getting dragged up, they're well looked after, and my house is clean … it's not a nice feeling that people are judging you when you're just trying to do your best.*” [Jessica, focus group 4].

In addition to discussing the potential negative emotional effects of public attitudes and stereotypes generally, participants also discussed how certain social situations can provoke feelings of embarrassment. Embarrassment appeared to be largey experienced in social situations where participants' financial or employment status became apparent to others, or where participants felt that there was a risk of being negatively judged by others. In one group, participants shared experiences of using free bus passes. Participants described how using these passes meant that “*the whole bus knows that … you're travelling free*” [Kevin, focus group 2], which was a source of embarressment because it was also seen to suggest to others that they were not in employment and received benefits. In the following extract, two participants discuss how they have avoided such situations as a means of managing anticipated stigma and embarrassment:

“*Do you know, once I went into town and I was kind of embarrassed to use it, I've actually paid.”* [Simon, focus group 2]

*“Because that's the way they make you feel, that you would rather pay so that they don't know that you're not working, so they don't know that you're getting certain benefits.”* [Kevin, focus group 2]

In another example of the feelings of embarassament some social situations can provoke, Hayley describes how she feels when somebody that she knows sees her at the Job Centre. These feelings are rooted in her beliefs about how others view people who are looking for work, suggesting that embarrassment stems from the risk of being judged negatively:

*“I always feel embarrassed going into the job centre … or even standing there and you see a person you know and you're like, oh my God, do you know what I mean? Because people look down on it.”* [Hayley, focus group 5]

In both of the previous examples, participants primarily attribute their feelings of embarrassment to other people, although it is also possible that these emotional responses result, at least in part, from their own beliefs and attitudes. In this sense, these emotional responses may also indicate a form of self-stigma.

## 4 Discussion

### 4.1 Overview of findings

The aim of this study was to identify aspects of poverty stigma that are most salient to individuals living on low incomes in Scotland, and may therefore contribute to health inequalities. Five themes were identified that reflect participants' experiences and perceptions of poverty stigma. At the structural level, participants described how the media produce stigmatising and unrealistic portrayals of poverty in the UK, while social security systems were deemed by participants to be degrading and unfair. Public stigma was evident in participants' descriptions of negative public attitudes toward people living in poverty, which were largely seen to be driven by both the media and a lack of understanding around the realities of living on a low income. Poverty stigma – and negative public attitudes in particular - was further seen to affect individuals’ self-esteem and produce feelings of negative affect and shame. These findings are broadly consistent with previous qualitative research conducted in other parts of the UK (Chase and Walker, 2012) and internationally (Reutter et al., 2009; Simons et al., 2018).

Structural stigma can be defined as institutional policies or practices that restrict individuals’ opportunities or wellbeing. One form of structural stigma emerges when institutions, such as media organisations, promote negative stereotypes about particular groups (Corrigan et al., 2004). Participants in the current study highlighted how “benefits shows” in particular promote stigmatising portrayals of people experiencing poverty, which is consistent with previous qualitative research in the UK (Chase and Walker, 2012). These programmes belong to a relatively recent genre of “Factual Welfare Television”, which is often criticised for propogating a number of negative stereotypes and in particular casting poverty as being the result of moral failings, such as laziness (De Benedictis et al., 2017).

These media representations likely have important consequences for people experiencing poverty and for wider society. People who read newspapers that promote relatively more negative portrayals of welfare claimants have been found to percieve higher levels of welfare fraud for example (Baumberg et al., 2012), which is consistent with participants’ views that media accounts shape public opinion. Rose and Baumgartner (2013) further argue that mass media are also an important driver of social policy, and demonstrate that increasingly negative portrayals of poverty in American newspapers is associated with later shifts toward reduced government spending for people living in poverty. Media coverage may therefore perpetuate both public and institutional forms of stigma, and erode support for public policies that might reduce economic and health inequalities.

A second form of structural stigma was evident from participants' accounts of claiming benefits. Participants described encountering stigmatising attitudes and behaviours from Job Centre staff for example, an issue that has also been highlighted by previous researchers (Chase and Walker, 2012; Patrick, 2016). A further issue highlighted was the demands that are made of claimants in order to receive benefits, percieved by participants as unfair and unrealistic. These accounts reflect the increasing use of behavioural conditionality in the UK, which requires claimants to undertake an increasing volume of work-related activities to demonstrate that they are actively seeking employment in order to qualify for benefits. (Dwyer & Wright, 2014). Failure to meet these requirements often results in a sanction, where claimants' benefits are stopped for several weeks or months. As previous researchers have noted, this use of behavioural conditionality frames unemployment as a matter of individuals' behaviour and ignores the economic and social factors beyond a person's control that may exclude individuals from the labor market. Recent evidence from Scotland has further highlighted the punitive nature of conditionality, where sanctions can be applied for relatively minor infractions such as attending Job Centre appointments a few minutes late (Wright et al., 2018). This research also demonstrates how sanctions can lead people to disengage with the social security system, or to experience increasing hardship and debt.

Participants also perceived members of the wider public as holding a number of negative attitudes regarding low income communities or neighbourhoods, which were thought to be largely derived from a lack of understanding among those who had little experience of financial hardship themselves. This indicates a form of percieved public stigma known as meta-stereotypes, which are defined as an individual's beliefs about how members of an out-group percieve and evaluate their own social group (Vorauer, Main & O’Connell, 1998). Negative meta-stereotypes have implications for health and well-being, even when individuals have not necessarily experienced overt discrimination or prejudice, and have been associated with lower self-esteem (Vorauer et al., 1998) and mental health outcomes (Jerald, Cole, Ward & Avery, 2017). It is important to distinguish here between meta-stereotypes and the actual prevelance of stereotypes amongst the public, although some data does suggest that negative attitudes regarding people experiencing poverty are relatively common; for example, results from the 2010 British Social Attitudes survey show that 23% of the public believe that poverty is caused by “laziness or a lack of willpower” (Scottish Government, 2015). An important area of future research will be to assess the relationship between population level attitudes and individuals' meta-stereotypes, and the joint effects of both on health outcomes.

Participants further discussed the potential cognitive, behavioural, and emotional consequences of stigma, which may reflect important pathways through which poverty stigma affects health. For example, lowered self-esteem was identified as being a potential response to negative public attitudes. This finding is particularly important, as longitudinal studies have shown that low self-esteem is a predictor of depression (Sowislo and Orth, 2013). Mickelson and Williams (2008) also demonstrated that self-esteem partially mediates the relationship between internalised poverty stigma and depression in a sample of low income women, further suggesting that low self-esteem is one pathway through which poverty stigma may influence health. Additionally, participants in the current study suggested that negative public attitudes – or meta-stereptypes – may become internalised by individuals, which in turn leads to lowered self-efficacy and aspirations for the future. A similar “why-try” effect is reported in psychiatric stigma literature, where individuals come to believe that they are either unworthy or unable of achieving personal goals after applying stereotypes to themselves (Corrigan et al., 2016). This discovery is a potentially important behavioural consequence of poverty stigma that warrants further attention.

It is important to note that participants' accounts of self-stigma and internalised negative attitudes largely referred to other individuals, and that there was little evidence that they themselves had experienced comparable effects on their self-esteem. This study highlights the complexity and naunce of stigma concepts, as participants appeared to distinguish between their *experienced* self-stigma, and *percieved* self-stigma (Pescosolido and Martin, 2015). Such a distinction invites further questions concerning individuals’ abilities to resist experiencing self-stigma personally, and the basis on which perceptions of self-stigma in others are formed.

Emotional reactions are a core component of stigma (Link et al., 2004), and participants reported experiencing negative emotions in response to percieved public attitudes especially. Feelings of embarrassment were highlighted in particular, which were often experienced in social situations that made participants’ social or financial situation apparent to others. These situations may represent instances of “identify threat”, which occurs when individuals believe they are at risk of being evaluated negatively by others. The feelings of embarrassment experienced in such situations are part of a broader psychobiological response to social self-threat that also includes inflammatory and hormonal changes, including increased cortisol activation (Dickerson et al., 2009). These physical changes can be adaptive as short-term responses to acute stressors, although chronic exposure to social threat can lead to dysregulation of these systems that in turn has negative implications for health (Dickerson et al., 2009; Juster et al., 2010). Identify threat processes may be especially important in the context of poverty stigma, as expereinces of poverty-based shame appear to be relatively ubiqutious (Walker et al., 2013).

Efforts to avoid shame or embarrassment may cause individuals to avoid accessing certain services. Some participants in this study discussed their reluctance to use free bus passes that people receiving certain disability benefits are entitled to in the UK. Other such examples have been highlighted elsewhere in the literature. In a recent UK survey, 23% of individuals who had claimed benefits in the past year reported that they have in the past either delayed claiming, or did not claim, benefits that they have been entitled for some shame-related reason (Baumberg, 2016). This included personal feelings of shame regarding claiming, a belief that they would be shamed by others, and an expectation that the process of claiming benefits would lead to shaming. Garthwaite (2016) also reports how feelings of shame and embarrassment can stop people from accessing foodbanks when in need, or delay applying for support from foodbanks until they reach a point of desperation, which exacerbates periods of food insecurity.

### 4.2 Opportunities for future research

The findings of this study highlight several aspects of poverty stigma that may have implications for health inequalities in Scotland and should be the target of future research. Previous researchers have argued that, “*essential to the scientific understanding of stigma is our capacity to observe and measure it*” (Link et al., 2004, p. 511), and considerable progress has been made in developing measures of various forms of stigma, most notably those related to health conditions such as mental illness (Brohan et al., 2010) and HIV (Earnshaw and Chaudoir, 2009). Such measures have been crucial in developing an understanding of how stigma affects health outcomes amongst patient populations, and in evaluating the success of stigma reduction interventions (Corrigan et al., 2012). Comparatively less progress has been made in developing measures of poverty-based stigma, however, which limits the ability of researchers to address similar questions in this field. An important step for future research will therefore be to develop new measures of poverty-based stigma in order to test how these experiences relate to population health within Scotland, and via which mechanisms. Reviews of health-related stigma measures show that such instruments are often multidimensional, and consist of multiple scales tapping different aspects of stigma (perceived stigma, self-stigma etc). The findings of this study suggest that a similar approach will be required when developing measures of poverty-based stigma. Despite the potential conceptual overlap between stigmatised identities or groups, it is unlikely that modifying an existing measurement model from another field can fully capture the experience of poverty-based stigma. Instead, measures of poverty-based stigma should be developed with a “bottom up” approach, where the item-content is developed to reflect the experiences and priorities of the target population (Terwee et al., 2007). The current study makes an important contribution in this regard.

Developing measurement models of poverty stigma will provide researchers with tools to test novel hypotheses regarding health inequalities, and potentially, to better understand spatial variations in health inequalities. A stigma-informed approach to health inequalities would suggest, for example, that the relationship between poverty and health outcomes will in part depend on the extent to which a society stigmatises those of lower SEP. In this sense, stigma may act as an effect-modifier that can – at least partly – account for some international differences in the scale of health inequalities (Popham and Boyle, 2011).

Measures of poverty stigma may also be helpful in better understanding the higher levels of mortality observed in Scotland compared to England and Wales that cannot be explained by differences in deprivation (Walsh, McCartney, Collins, Taulbut & Batty, 2017). This excess mortality may be partly attributed to the inadequate measures of deprivation in the UK, which do not adequately capture the “lived reality” of poverty (Walsh et al., 2017). Further developing an understanding of stigma may help to inform the measurement of these lived experiences, as well as exploring any potential regional variation.

The results of this study have implications for policy and practice, and there is now scope to consider how interventions might be designed to reduce the prevalence of poverty stigma within Scotland and elsewhere. A whole-system approach that simultaneously addresses stigma at various structural, public, and individual levels is necessary (Link and Phelan, 2001), and may include, for example, redesigning public services, challenging negative media representations, and social marketing campaigns targeting public attitudes.

### 4.3 Strengths and limitations

A major strength of the qualitative approach employed in this study is that it enabled a rich exploration of the experiences and perceptions of poverty-based stigma that are most salient to individuals with experience of living on a low income in Scotland. Despite its strengths, he study does also have limitations; in particular, the findings are based on the views and experiences of a small group of ethnically similar individuals living in urban areas. The findings therefore cannot be considered to be representative, and individuals from black minority ethnic backgrounds and those living in rural areas or the islands of Scotland in particular may have different experiences of poverty stigma. Similarly, it was beyond the scope of this research to consider issues of intersectionality. This is an important limitation, as several social groups who disproportionately experience poverty in Scotland – such as those with disabilities and ethnic minorities - may experience multiple forms of stigma or discrimination in addition to poverty-based stigma.

## 5 Conclusions

Poverty based stigma is a potentially important determinant of health inequalities, although little is known about this topic from a Scottish perspective. Participants' accounts in this study reflected several forms of poverty-based stigma operating at different structural, social and individual levels. Structural stigma was evident in participants' descriptions of how poverty is portrayed in the media and in their experiences of social security systems, whilst public stigma was evident from participants’ perceptions of social attitudes regarding poverty. Participants further discussed the emotional consequences of experiencing stigma, which included feelings of embarrassment. It was also suggested that individuals may internalise negative public attitudes, although there was little evidence that participants had done so themselves. These various forms of stigma may influence population health, and there are a number of plausible societal and individual-level mechanisms through which this could occur. A major challenge for future research will be to develop measures of poverty-based stigma, and to examine how these processes relate to health outcomes, and via which mechanisms. Working to challenge poverty stigma in all of its forms will complement current anti-poverty strategies and may be a promising new approach to improving population health and reducing health inequalities.

## Funding

This work was supported by the Scottish Collaboration for Public Health Research and Policy core grant from the Medical Research Council and Chief Scientist Office of Scotland [MR/K023209/1]. The funders played no role in the conceptualization or realization of the research and no role in the decision to submit it for publication.
