# Supplementary material for: Health inequality implications from a qualitative study of experiences of poverty stigma in Scotland
Source: Soc Sci Med. 2019 Jul;232:43–9. doi: 10.1016/j.socscimed.2019.04.033 (PMC6597943; doi:10.1016/j.socscimed.2019.04.033)
Supplement: Multimedia component 1 [file mmc1.docx]

Supplementary Material 1: Topic Guide

| **Introduction**   - Thank everyone in the group for attending. - Briefly cover the background and aims of the study. Manage expectations of what research can achieve and what it can change. Emphasise people can leave or stop participating at any point. Field any questions. - Set sound ground rules for the focus group and allow participants to suggest their own rules: e.g. being respectful of all views expressed, not repeating anything that’s said in the room. |
| --- |
| **Main discussion**   - **What does poverty in the UK and stigma mean to me?**   - Ask the group to write down ideas on cards, then display these and discuss as a group   - Try to get a sense of how the group think about stigma and poverty – establish a shared meaning to make sure everyone is on the same page   - If there’s less discussion of stigma, introduce prejudice and discrimination which might be easier to relate to. - **What do you think are some of the general public attitudes around poverty?** - **What do others think about people who live in poverty, or struggle on a low income?**    - Try to open the conversation up broadly, and steer people toward discussing public and perceived stigma.   - Discussion prompts:     - are there any stereotypes around people who live in poverty?     - what do people think causes poverty?     - how is poverty discussed in the newspapers and television?     - do you think other people know what it’s like to live in poverty? what do they think, and where do they get it wrong? - **Are there any circumstances or situations where you think people who live in poverty or live on a low income might be treated unfairly or poorly by others?**    - Trying to get at feelings of anticipated stigma here, and also make it easier to discuss personal experiences of discrimination. Personal experiences might fall out naturally.     - E.g.: going out shopping, being out and about or at work. - **Does anybody here have any experiences of being treated unfairly or poorly because of their financial situation?**   - Could use any examples raised previously as discussion prompts. - **Reflecting on some of the things that we’ve spoken about today, how do you think these experiences affect people?**   - Use examples that have come up from public stigma, anticipated stigma and experienced stigma   - People may raise issues of self-stigma here: thinking less of themselves as a result of others’ attitudes or behaviours, and feeling emotions like shame.     - Explore these issues further if raised. |
| **Close**   - **Thinking back on the issues that we’ve discussed today, what one thing would you like to see change if you had a magic wand?** - **Is there anything else that anybody would like to raise?** - Thank the group for taking part and acknowledge that this is a difficult topic to speak about. - Distribute debrief sheets. - Reiterate what will happen next with the data, and how we will be planning to share the results in reports and presentations. |
